# Supplementary material for: Emerging many-to-one weighted mapping in hippocampus-amygdala network underlies memory formation
Source: Nat Commun. 2024 Oct 26;15:9248. doi: 10.1038/s41467-024-53665-9 (PMC11513146; doi:10.1038/s41467-024-53665-9)
Supplement: Supplementary file 1 — Supplementary Information [file 41467_2024_53665_MOESM1_ESM.pdf]

Supplementary Figures (Liu, Hall, & Wang)

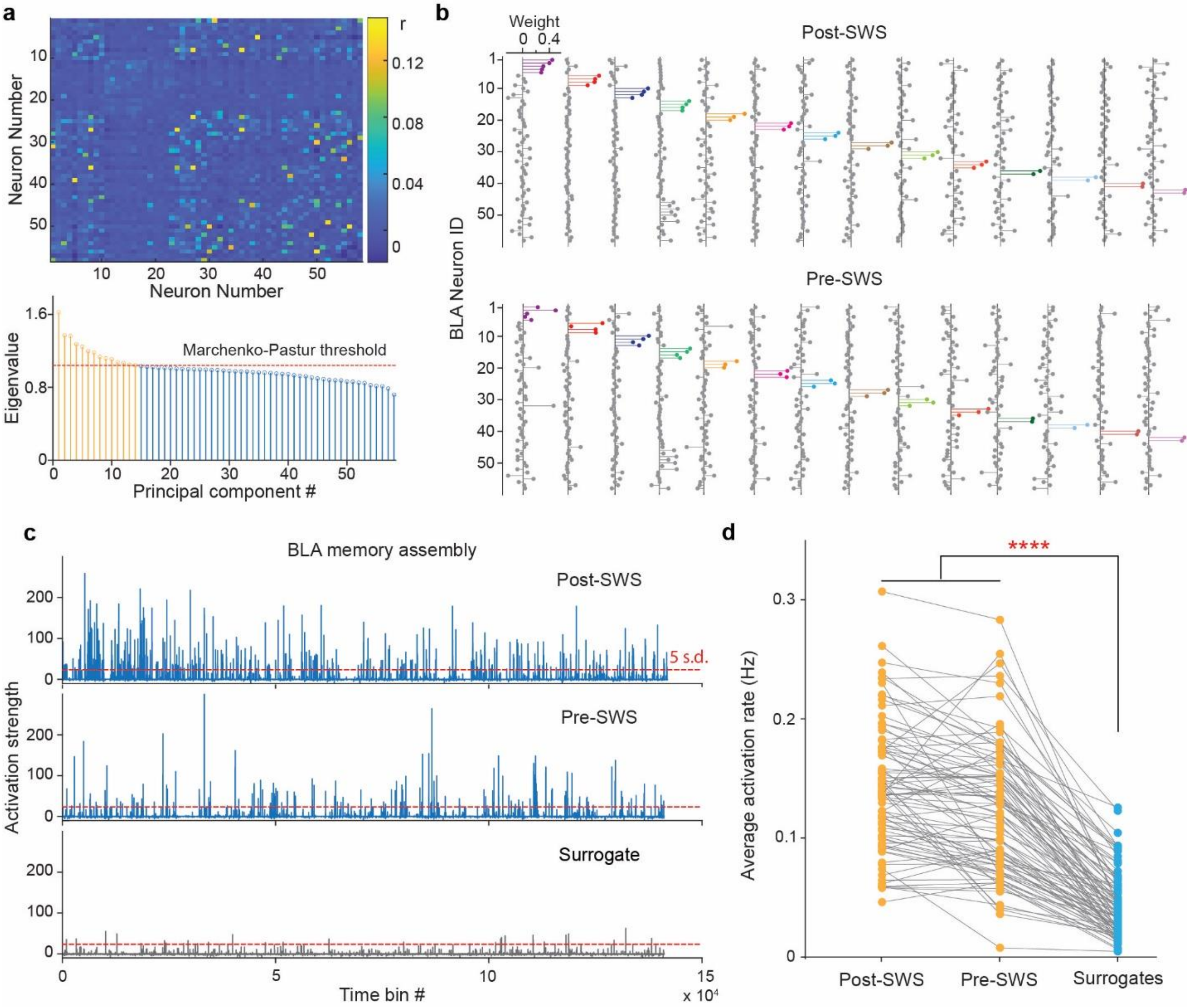

Suppl. Fig. 1: Independent component analysis (ICA). a, Correlation coefficient map of 58 BLA neurons (top; the same as shown in Fig. 1d) and eigenvalues of the principle components (bottom; Marchenko-Pastur threshold was used to determine the number of assemblies). **b,** ICA largely identified the same 14 assemblies based on separate datasets (pre-SWS *vs.* post-SWS; neurons are the same as shown in Fig. 1d). Neurons are arranged in the same order in top and bottom panels. **c,** Activity strength of assembly 2 (as shown in Fig. 1d) during pre- and post-training sleep and surrogate. **d,** Activation rates of all identified BLA assemblies (n = 88 from 10 mice) are significantly higher than chance during both pre- and post-training sleep. $P < 0.0001$, $F_{1.860,\ 87.42} = 208.6$, one-way ANOVA; ****$P < 0.0001$, Bonferroni post-hoc.

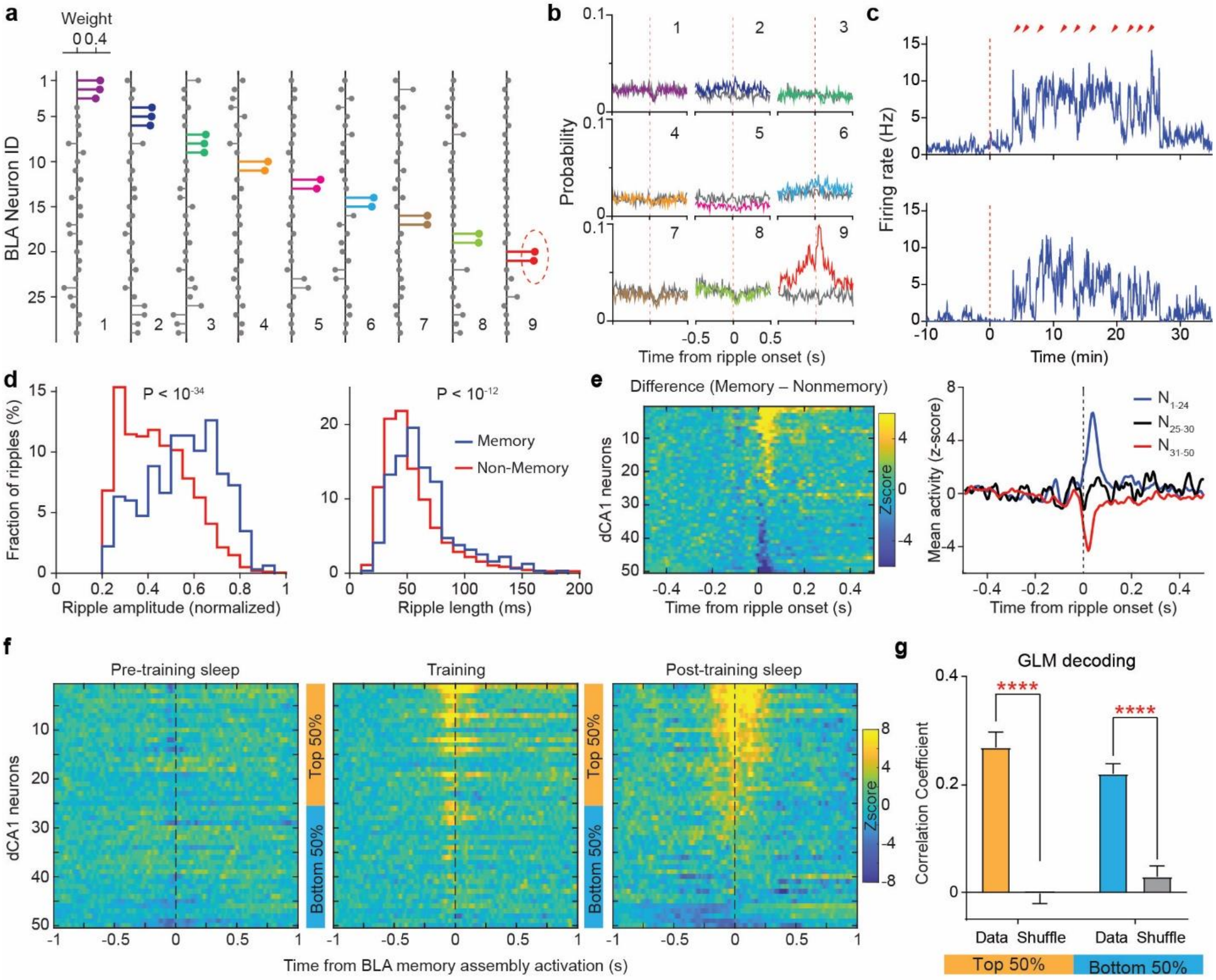

Suppl. Fig. 2: Emerging many-to-one weighted mapping underlying memory formation (Mouse #2). a, ICA identified 9 assemblies based on spikes of 29 BLA neurons recorded during post-training sleep. **b,** Cross-correlograms between dCA1 ripples and the 9 BLA assemblies. Grey and color lines indicate pre- and post-training sleep, respectively. Note: assembly 9 was characterized as BLA memory assembly. **c,** Rate histograms of the two BLA memory neurons (assembly 9 as shown in panel a) during contextual fear training. **d,** Memory-associated ripples have significantly larger amplitude (left) and longer duration (right). **e,** Left, activity difference of dCA1 neurons (recorded simultaneously with the BLA neurons as shown in a) in relation to memory *vs.* non-memory ripples (see Fig. 3d for details). Right, mean activity of upregulated (#1–24), unmodulated (#25–30), and downregulated dCA1 neurons (#31–50). **f,** Cross-correlogram heatmaps between the BLA memory assembly and dCA1 neurons during the pre-training sleep (left), training (middle), and post-training sleep (right). Neurons are arranged in the same order in the three heatmaps. **g,** GLM decoding indicates that both the top 50% (corresponding to neurons #1–25 as shown in panel f) and bottom 50% dCA1 neurons (#26–50) predict the firing rates of the BLA memory assembly, in comparison to the shuffled data (repeated 100 times).

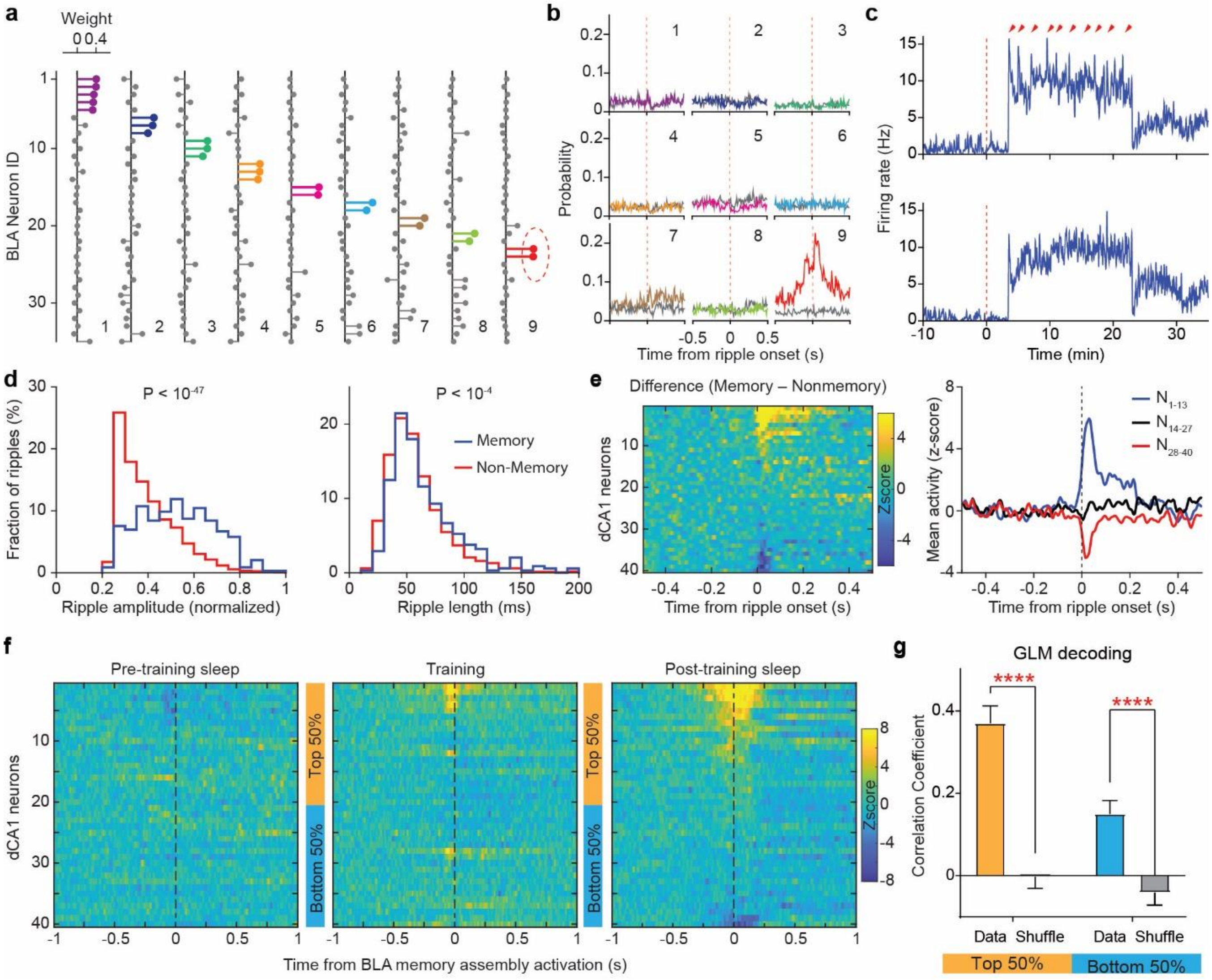

Suppl. Fig. 3: Emerging many-to-one weighted mapping underlying memory formation (Mouse #3). a, ICA identified 9 assemblies based on spikes of 35 BLA neurons recorded during post-training sleep. **b,** Cross-correlograms between dCA1 ripples and the 9 BLA assemblies. Grey and color lines indicate pre- and post-training sleep, respectively. Note: assembly 9 was characterized as BLA memory assembly. **c,** Rate histograms of the two BLA memory neurons (assembly 9 as shown in panel a) during contextual fear training. **d,** Memory-associated ripples have significantly larger amplitude (left) and longer duration (right). **e,** Left, activity difference of dCA1 neurons (recorded simultaneously with the BLA neurons as shown in a) in relation to memory *vs.* non-memory ripples (see Fig. 3d for details). Right, mean activity of upregulated (#1–13), unmodulated (#14–27), and downregulated dCA1 neurons (#28–40). **f,** Cross-correlogram heatmaps between the BLA memory assembly and dCA1 neurons during the pre-training sleep (left), training (middle), and post-training sleep (right). Neurons are arranged in the same order in the three heatmaps. **g,** GLM decoding indicates that both the top 50% (corresponding to neurons #1–20 as shown in panel f) and bottom 50% dCA1 neurons (#21–40) predict the firing rates of the BLA memory assembly, in comparison to the shuffled data (repeated 100 times).

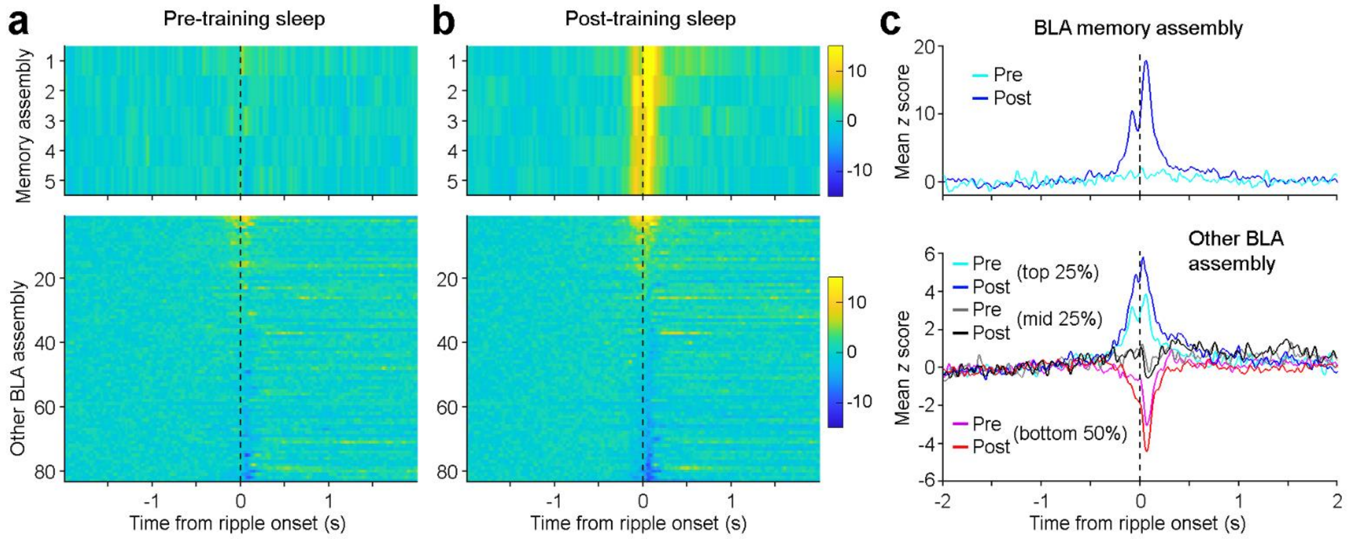

Suppl. Fig. 4: BLA assemblies display dCA1 ripple-correlated activity. a, Heatmap activity of individual BLA memory assemblies (top) and non-memory assemblies (bottom) in relation to dCA1 ripple events during pre-training sleep. **b,** the same as a except showing post-training sleep data. Assemblies are arranged in the same order in a and b; color bars indicate *z* score. **c,** Mean activity of BLA memory assemblies (top) and three subsets of BLA non-memory assemblies (bottom) in relation to dCA1 ripple events during pre- and post-training sleep. The top 25%, mid 25%, and bottom 50% indicate assemblies #1–21, #22–42, and #43–83, respectively, as shown in panels a/b (bottom).

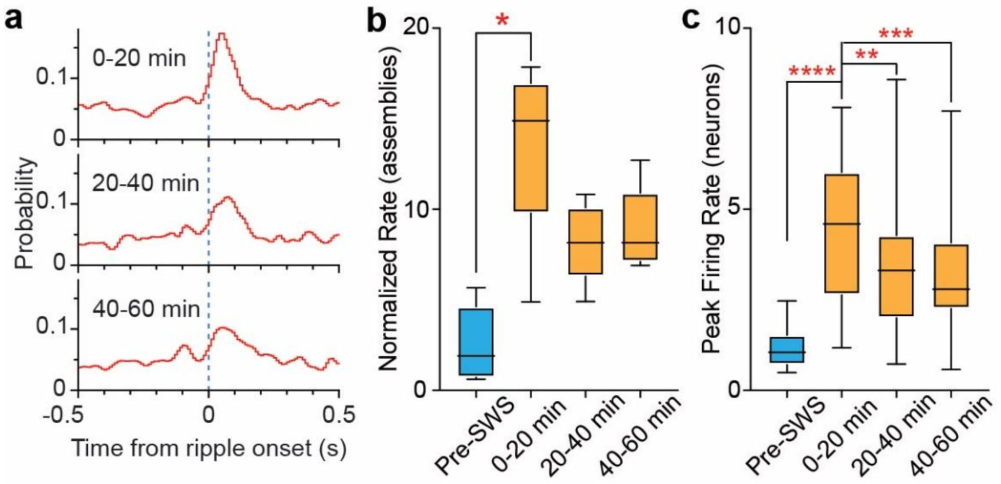

Suppl. Fig. 5: A gradual decay of dCA1-to-BLA communication during post-training sleep. a, Cross-correlograms between dCA1 ripples and BLA assembly 2 (as shown in Fig. 1e) across post-training 20-min sleep epochs. **b&c,** A gradual decay in the correlation strength between dCA1 ripples and BLA memory assemblies (n = 5; b) or BLA memory neurons (n = 18; c) across the post-training sleep epochs. $P < 0.01$, $F_{1.530,\ 6.121} = 13.84$ (b) and $P < 0.0001$, $F_{1.391,\ 23.65} = 31.28$ (c), one-way ANOVA; *P <0.05, **P <0.01, ***P <0.001, ****P <0.0001, Bonferroni post-hoc.

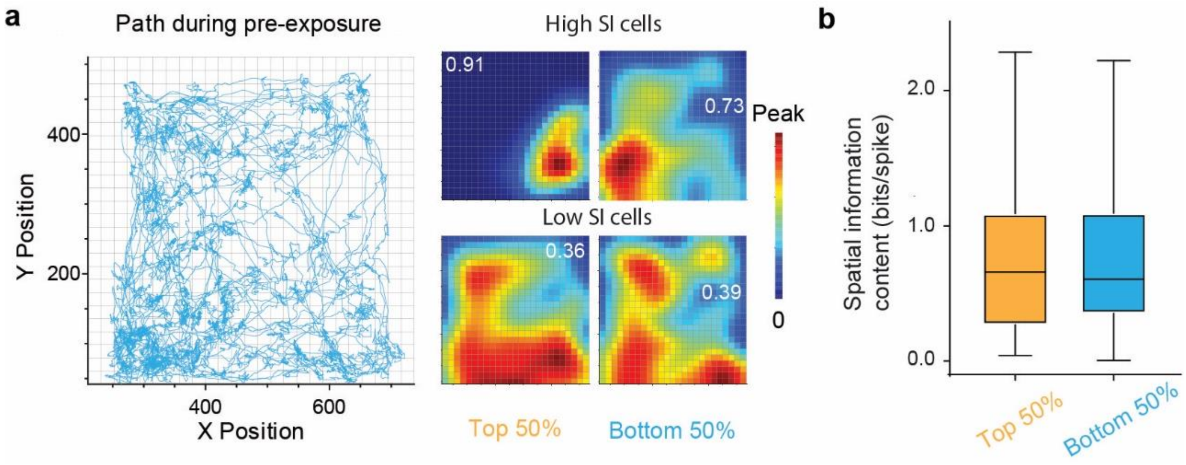

Suppl. Fig. 6: Spatial information analysis of dCA1 neurons. a, A representative navigation path (left) and firing rate heatmaps of 4 example dCA1 neurons recorded simultaneously (right). Top 50% and bottom 50% are the same as that defined in Fig. 5; numbers in each heatmap indicate information bits per spike. SI, spatial information. **b,** There is no difference in information content between the two subgroups of dCA1 neurons (P = 0.774, unpaired *t* test).

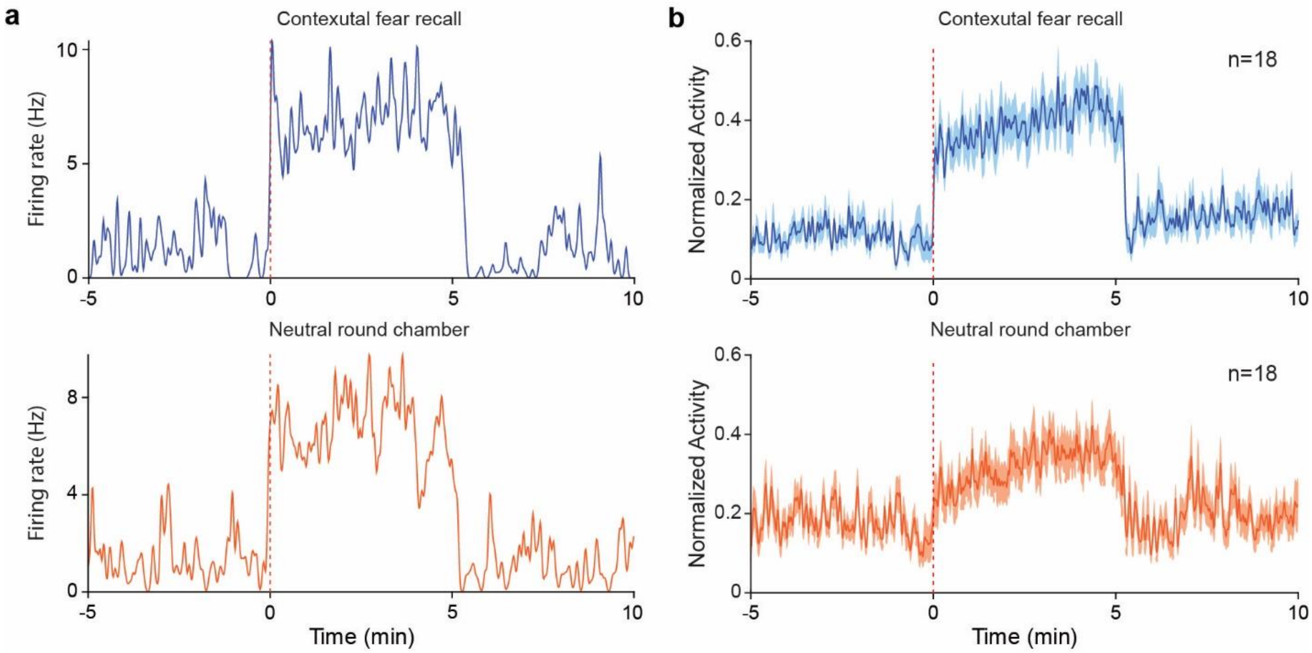

Suppl. Fig. 7: Generalized responses of BLA memory neurons to neutral contexts. a, Rate histograms of one representive BLA memory neuron upon exposure to the shock chamber (top) and a novel neutral chamber (bottom) after contextual fear training. **b,** Mean activtiy (± s.e.m.) of all BLA memory neurons upon exposure to the shock chamber (top) and neutral chamber (bottom) after contextual fear training. Normalized activity is defined as the firing rate divided by the peak fring rate, where the peak firing rate is the highest firing rate of each neuron across all bins during contextual fear recall (bin size: 1 s).
